# Failed surrogate conceptions: social and ethical aspects of preconception disruptions during commercial surrogacy in India

**DOI:** 10.1186/s13010-016-0040-6

**Published:** 2016-09-19

**Authors:** Sayani Mitra, Silke Schicktanz

**Affiliations:** 1 Department of Medical Ethics and History of Medicine, University Medical Center Göttingen, Humboldtallee 36, Göttingen, 37073 Germany; 2 Göttingen Centre for Gender Studies, Humboldtallee 36, Göttingen, 37073 Germany

**Keywords:** Commercial surrogacy, Missed/failed conceptions, Preconception disruptions, Loss, Care ethics, India

## Abstract

**Background:**

During a commercial surrogacy arrangement, the event of embryo transfer can be seen as the formal starting point of the arrangement. However, it is common for surrogates to undergo a failed attempt at pregnancy conception or missed conception after an embryo transfer. This paper attempts to argue that such failed attempts can be understood as a loss. It aims to reconstruct the experiences of loss and grief of the surrogates and the intended parents as a consequence of their collective failure to conceive a surrogate pregnancy.

**Methods:**

Drawing on a qualitative study conducted over a period of eight months between 2014 and 2015 at two fertility clinics in Delhi and two in Kolkata, India, this paper examines the experiences of the surrogates and the intended parents when faced with missed conceptions or failed conceptions during a surrogacy arrangement.

**Results:**

We argue that while the surrogate grieves the non-arrival of a ‘good news’ as an uncertain loss, the intended parents experience yet another, failure in addition to the losses they might have incurred during their previous fertility treatments. The body of the surrogate becomes a site of ‘a lost opportunity’. The surrogate embodies a loss in her quest to achieve social mobility and the intended parents experience a disembodied pregnancy loss. This very emotional experience stands in stark contrast to the conceptualisation of such failed attempts as non-events within the discourse of the surrogacy industry. The experience of loss of the intended parents is recognised but their grief is given no space. We argue that such ambiguity around the nature of losses resulting out of a missed or failed conception during surrogacy is an outcome of lack of interpersonal relationship between the surrogate and the intended parents.

**Conclusions:**

Since commercial surrogacy is a relational process, the only way in which the experiences of losses and failures of the actors at the preconception stage can be better addressed is through developing close sharing and understanding between each other through an *ethics of care*. Therefore, to nurture caring relationships, surrogacy needs to be understood as a moral commitment by –the surrogates and intended parents. To enable such a commitment, there is a need to reconsider the pre-defined and legally regulated professional duty of the doctors, agents and agencies. It cannot be a one-sided commitment, but has to have elements of mutuality.

## Background

The technology-guided practice of commercial gestational surrogacy is highlighted both within the medical and the public discourse as a means towards achieving reproductive successes. Surrogate conceptions have been described by scholars as conceptions having its genesis in the heart [1]. These conceptions take place much ahead of its materialisation, in the minds of the actors through their continuous planning, strategising and participation during the actual preconception phase. This includes the process of hormonal stimulation, conducting vaginal ultrasounds, or even embryo fertilisation. Therefore, the hopes, dreams and expectations of the actors participating in the process of gestational surrogacy are closely intertwined with the complex process of technological intervention. Since technology is enacted on the body of the surrogate, her body becomes a site of hope and ‘surveillance’ [2]. However, amidst this dominant narrative of success and optimism (see also [3]), a narrative of failure is often neglected or not discussed.

One of the main reasons for such a one-sided narrative is the faith of the key actors- the surrogates and the intended parents on assisted reproductive technologies. Quality medical healthcare in India is largely concentrated in the hands of the economically privileged (see also [4]). Lack of access to medical technology, especially reproductive technology during their own pregnancies, prompts the surrogates to understand ‘technology’ as a powerful force that is outside their grasping capacities. However, since quality [read private sector] heath care facility and use of medical technology during pregnancy is considered the domain of the privileged class in India due to its unaffordability by its lower income groups (refer to [5]); the Indian surrogates perceive assisted reproductive technologies (ART) as a tool of modernity. Despite initial apprehension, the surrogates repose full faith in medical expertise and hope for success. The intended parents are increasingly attracted to the option of surrogacy since the practice is marketed by its clinics as an easy solution to their problems of infertility and childlessness [6] through strategic advertising, marketing and packaging schemes offered by these clinics. Such factors have played a key role towards an expansion of market in commercial surrogacy in India leading to an annual turnover of $ 4 million [7] with its expanding transnational and domestic market until the practice got banned for foreigners in 2015. This trend of rapid spread and popularity of the industry of commercial surrogacy in India has also been studied by ethnographers (refer [8–11]).

In this paper, we discuss the experiences of the surrogates when they fail to conceive a pregnancy and the impact of the same upon the intended parents. To present this narrative of failure, we analyse the nature of loss of both the actors and their struggle for a space to express themselves or grieve. Our aim is to explore the ways in which such experiences of missed conceptions can be offered a better recognition within the surrogacy discourse. In particular, we argue that relationships between the actors involved in surrogacy can be strengthened so that the actors themselves are able to support each other during such events. Based on this assumption, we examine the manner in which this can be achieved. By doing so, we seek to contribute to the larger question of how improved communication and a better support system can empower the lay actors in dealing with the risks and losses resulting out of the use of ARTs. While taking about risks and disruptions, it is important to distinguish between the pre-conception and post-conception reproductive failures taking place during surrogacy. While the pre-conception failures involving missed conceptions or chemical pregnancies are commonplace and are something which the actors are aware of, even if not prepared to face, the post-conception disruptions involving miscarriages, foetal reductions and selective abortions follow a different trajectory. In this paper, we focus only on the first type, because the second type requires a different kind of ethical analysis that is being discussed in another paper.^1^

## Background

Surrogacy is not a 100 % reliable procedure despite its popularity and contrary to what the advertisements of surrogacy clinics might suggest. According to Society for Assisted Reproductive Technology’s Report 2008, out of the 2502 gestational surrogacy cycles performed at the reported clinics, only 39,45 % of cycles were successful in terms of live births leading to 987 gestational births and 1395 gestational surrogacy babies [12]. The 2013 data for clinics in the U.S.A., show that out of the reported cycles performed for gestational carriers with patient oocytes (of ages below 35), 46 % of those cycles failed [13]. This clearly indicates that not all embryo transfer performed towards achieving a surrogate pregnancy results in a success.

The procedure of surrogacy begins with stimulating the body of the surrogates with estrogen and progesterone, in order to make her uterine lining receptive to an embryo transfer (ET). During the fieldwork, we noticed that the procedure of ET formalises a surrogacy arrangement with signing the contract between the surrogate and her husband^2^, and the intended parents along with disbursal of the first payment installment. Usually 12–14 days after an ET, a *betaHCG test* of the surrogate is conducted to confirm pregnancy. If the result is positive, an ultra-sonographic scan of the surrogate is conducted two weeks later to re-confirm pregnancy and dismiss all chances of chemical pregnancies.^3^ But if the test result is negative due to failure of the embryo to implant, the actors are informed about the result and all medications are stopped (see Table 1 for a temporal understanding about the preconception stage of surrogacy).

**Table 1:** Stages of surrogacy preconception and possible disruptions (Source: Author’s Research)

Process of surrogacy: preconception	Labour involved for the surrogates	Possible reproductive disruptions
Screening of surrogate (and donors)	Frequent visits to clinics for ultrasonography (vaginal and abdominal) and blood tests, hormonal shots & medicines	Need for re-matching
Hormonal stimulations		
Intended parents & surrogate (& donor) matching		
Ovum pickup &/or sperm collection (if needed)		
Fertilisation & incubation		
Embryo freezing (usually)		
Signing of contract		
Embryo transfer	Embodiment of embryo, movement restrictions, hormonal shots	
Waiting period	Embodiment of embryo, movement restrictions, hormonal shots	
Pregnancy test (betaHCG)	Blood test followed by abdominal ultrasonography	Missed conceptions, chemical pregnancies

Lynda Layne (2007) when writing about women-centered approach on pregnancy loss states that “women who labour deserve to be treated with dignity regardless of whether their labor will result in a live birth or not. In order to accomplish this, we need to understand the special physical and emotional needs of women undergoing miscarriage or stillbirth” ([14], p. 94). Such experiences are common for women undergoing IVF assisted pregnancies. Infact couples undergoing failed IVF attempts are deeply impacted by their failures, given their history of unsuccessful attempts at childbirth. Expanding this chain of thought, we would like to argue that just like miscarriage or stillbirth, failures during the preconception stage also deserves attention. By ‘preconception’, we are referring to the medicalised definition of preconception within the ART discourse where a pregnancy conception takes place through the conscious act of transfer of fertilised embryo(s) into the uterus. This encompasses the entire preparatory phase involved during commercial surrogacy including screening, selection, tests, fertilisation, and transfer. All stages contributing to the materialisation of a pregnancy conception are referred to as preconception stage.

## Methods

The empirical data used in this paper was collected through a qualitative study conducted over a period of eight months between August 2014 and May 2015 at two clinics in Delhi/NCR^4^ in the Northern part of India and two in Kolkata in the Eastern part of India. Ethical approval for the study was obtained in two steps, the first from the Ethics Commission of University of Göttingen and the other one at the site/country of study from a designated ethics committee at the Delhi University.

The initial period of access to the field was difficult and very time consuming because of gate-keepers obstructing entry and access at every potential point of contact (see [15]). Especially after recent media reports in 2014 on a surrogacy scam in Thailand [16] where Australian commissioning parents abandoned a twin born with Down’s syndrome, it became all the more difficult for any outsider to build trusting relationships and gain access to the field. However, after repeated requests, access became available. The study was multi-sited as it was conducted in surrogacy clinics, surrogate homes, surrogacy agency offices, public places and homes by employing methods of semi-structured in-depth interviews [each lasting on an average of 25–40 mins], non-participant observation and case studies (see Table 2). The intended couples interviewed were of Indian origin residing in India and overseas, heterosexual and married.^5^ Non-participant observation was mainly conducted at the waiting rooms of the fertility clinics, at the seating area of the clinical staff, seating area of the surrogates and occasionally during consultations between doctors and the intended parents. Notes were taken down during the observation and personal reflections were added to it later to fill in the gaps. Those interviews and encounters with surrogates and intended parents which were rather detailed and went beyond the stipulated set of questions and time, reflecting the structures of the surrogacy industry through a detailed narrative of their own lives, expectations and experiences, were taken as case studies. A case study is usually seen as an instance of a border phenomenon, as part of a larger set of parallel instances’ ([17], p. 2). While six of these cases were ‘typical cases’ and provided insight into the regular working of the surrogacy industry, two were ‘atypical cases’ suggesting the possibility of rather unconventional relationship between the surrogates and the intended parents ([18], p. 217) Prior written informed consent^6^ was taken from all respondents. The empirical data collected was manually transcribed and translated from Hindi and Bangla to English. The analysis of the data was done using the method of content analysis [19, 20] and all respondents were pseudonymised. Hence all names mentioned in this article are pseudonyms.

**Table 2:** Research Methods and Multi-sited Sample Size: Location Codes: Clinics 1 – 5 in Delhi = C1 – C5; Clinics 6 – 7 in Kolkata = C6 - C7; Surrogate Homes 1–2 in Delhi = H1- H2

S. No	Research techniques	Category of respondents	Sample size	Location-wise distribution of sample	
1	Semi structured in-depth interviews	a) Surrogates	45	C1	5
				C2	6
				C3	11
				C6	15
				C7	3
				H1	1
				H2	4
		b) Intended parents	15	C1	7
				C2	5
				C6	2
				C7	1
		c) Fertility doctors	5	C1	1
				C2	1
				C4	1
				C5	1
				C6	1
		d) Agents (local agents and from agencies)	7	C1	1
				C3	1
				C6	4
				H1	1
		e) Clinic staff	4	C1	1
				C3	2
				C6	1
		f) Surrogate home staff	1	H1	1
2	Non participant observation	IVF clinics in Delhi (including the National Capital Region/)	2	H1	1
				H2	1
3	Case studies	a) Surrogates	*5*	C2	1
				C3	1
				C6	3
		b) Intended parents	3	C1	2
				C6	1

Source: Author’s research

Our initial field experiences suggested that the surrogacy industry in India had widened its horizon to serve not just the heterosexual, married foreign nationals and Non-Resident Indians (NRIs), but also catered to the emerging domestic demands of its own middle classes (as also confirmed by [21]). Hence we decided to concentrate mainly on commercial surrogacy arrangements commissioned by heterosexual couples of Indian origin either residing in India or abroad, to be able to capture the Indian experience in the working and spread of the industry. Incidentally few months after the commencement of the fieldwork, the Indian government prohibited commercial surrogacy for foreigners but continued keeping the market open for its own citizens, Non-resident Indians (NRIs), Persons of Indian origins (PIs) or even for couples where one of the spouses is an Indian citizen or is of Indian origin. This makes the context of our research even more suitable and timely [22].

Although the research question with which the field was approached was very different and aimed towards looking at the subject formation of the foetal entity, the themes of failure especially after a first attempt at a surrogate ET stood out from the interview responses. Further, despite their preparedness for the same at the commencement of the process, a theme of loss was constant within the accounts of those respondents having experienced missed or failed conceptions. To examine the reasons of the same, it seems important to capture the experiences and understanding of the surrogates and the intended parents regarding surrogacy and pregnancy particularly during the preconception stage.

## Results and discussion

The surrogates and the intended parents experience different variants of losses and find their own ways of constructing meaning of those, when faced with events of missed or failed conceptions. Within the clinical discourse, such failed attempts to surrogate pregnancies are not considered as events of any significance. Their occurrence being very common (as mentioned above) — they are normalised by maintaining silence. The intended parents and surrogates are broadly aware of the chances of the failed cycles. However, what becomes important to observe is the impact of these failures or missed attempts to attain a surrogate pregnancy on them. In the following sections, we reconstruct their experiences of loss and their inability to grieve by analysing the experiences, expectations and the nature of relationships between the actors during surrogacy. We further analyse the causality of such sense of losses, its bearing and impact upon the actors. By doing so, we argue in favour of a due recognition of such preconception losses and bring out the significance of these missed or failed conceptions within the surrogacy discourse.

### Embodying an ‘opportunity’

Group of thirteen potential surrogates sat at the reception of an IVF clinic in Delhi waiting for their first ultrasonography scan. One of them named Meera started talking to me and said:

*“… My heart is beating so fast. Don’t know what they will do inside [the doctor’s chamber]. They told me that the child will be someone else’s. If I can give them their child from my womb, they will give money. Women in our neighborhood have done it and have got money. This work is better than doing ‘beldari’ [digging and carrying soil and cement] …”*

Meera’s words reflected that surrogates decide to take up a ‘chance’ or ‘opportunity’ available in front of them to embark upon - what they understand as an unconventional labour, in order to earn a secure future and to materialise the intended parents’ reproductive aspirations. By doing so, they attempt to embody an ‘opportunity’ coming their way. Their reproductive potential becomes their identity and strength. Their body becomes a site where reproductive aspirations, technological expertise and hopes are enacted. While on one hand, the technologies of visualisation, monitoring and regulation like the ultrasonography, regular blood tests, urine tests have brought their bodies under a medical gaze; on the other hand, the innate processes of their body, the unpredictability of the technological interventions and the wait for the results prevent the outsiders from casting a complete control over their body. This unpredictability to some extent keeps the mysticism around pregnancy alive. To establish a better control over the situation, the clinical discourse constructs the body of the surrogates to be ‘at risk’- which has been a typical tendency within the field of biomedicine [23]. The surrogates are thus made to feel responsible for the well-being of themselves and the aspirations of every other actor involved in the procedure of surrogacy. The surrogates weigh the risks and benefits presented in-front of them by the agents and submit themselves to the procedure. From the moment of induction, the agents and the clinical staff continuously remind the surrogates about their duty towards taking good care of their bodies and taking medication and injections on time. A very common statement which agencies are heard making to their surrogates is, *“…You have to give up on your spicy food habits for the ‘thing’ growing inside you”.* By entering into a surrogacy arrangement, the surrogates let the outsiders (agents, physicians, nurses, intended parents) into the most intimate details of their life including their sex lives, reproductive histories and family history of ailments. From the day of ET till the next two weeks, the local agents and/or the agencies keep the surrogate under strict vigilance. During this period, surrogates are either housed in a surrogate home if such an arrangement is available; or in a nursing home or guest house in cases where the surrogate is later expected to complete the pregnancy by staying at their own home. They are instructed to maintain an emotional distance from the embryonic entity that they are expected to bring into existence. They are asked to *“think positive and be happy”* since their ‘emotional wellbeing’ might have an impact on their conception. After the ET, an intended mother named Kushboo told her surrogate: *“… Please eat well and do not lift anything heavy and take rest as much as you can. Don’t eat rich food and take medicines on time…”* Such concerns and advices are commonplace within the surrogacy narrative.

The surrogates follow the mandates of a routinised life rested on them with the hope of earning a secure future for themselves and their families. However, even if they submit their bodies to technological intervention, their reproductive capacity becomes their strength and provides them with a sense of control. Having gestated their own pregnancies, the surrogates feel that they know and understand their own bodies. A potential surrogate Radha at a clinic in Delhi while she waited for her first ultrasonography to confirm her suitability for the job said: *“… I am not infertile. I have my menses. Why shouldn’t I get pregnant…?”* They become confident of being able to complete a surrogate pregnancy successfully. By doing so, they start hoping - maybe as strongly as the intended parents - for a successful conception and pregnancy. They rationalise in favour of obediently following all prescribed rules, restrictions and instructions about taking medications and ‘caring’ for their ‘body’ by keeping it safe from any impeding physical risk. They embody a risk and try to dispel any other further risks from coming their way and displace their efforts.

The interviewed surrogates considered the task of successfully completing their surrogate pregnancy as their immediate goal and wished to convert this ‘window of opportunity’ into a significant achievement in life. An aspiring surrogate in Delhi, Alka once said: *“ …To achieve something in life, women have to get out of home and do what they are good at, without caring about the world…”.*

However, we found that a disruption in the desired course of action in the form of an unsuccessful surrogate conception shakes their confidence, which brings them a sense of loss, causing grief. A surrogate in Kolkata named Gopa who had a failed pregnancy a month before mentioned that:

*“…I and my husband were so hopeful. My husband was more confident than me. I have never had a tad bit of a complication during my two pregnancies. Both my sons were born out of a normal (vaginal) delivery. Don’t know why this didn’t work out. Perhaps it’s just my bad luck…”*

The news of failure comes to them as a big setback. As a surrogate Bharati at Kolkata while sharing her failed pregnancy said:

*“…I was very upset when the attempt did not succeed. I was very sad and used to cry a lot, the particular party never came back, but the doctor did not give up on me. He tried to help me become surrogates for other parties, but when those attempts failed too, they asked me to work for them to recruit others, as in that way I would be able to help myself too…”*

The waiting period between an ET and a pregnancy test gives the surrogates some time to hope for a better future and dream about their upcoming nine months, especially for those who are made to stay at the surrogate homes or at a private nursing home or apartment during the waiting period. A change of place and/or interactions with fellow pregnant surrogates makes them long for these pregnancies even more. But a missed conception disrupts their plans by holding the chances of placing them back to their old life, from where they had aspired to outgrow.

### The non-existent loss

The surrogates are neither able to comprehend the medical reason behind their failed pregnancies nor are they sure about the nature and extent of the loss. Therefore, we would like to define their loss that they experience as an “ambiguous or uncertain loss” [24]. What they figuratively lose in this context are “elusive embryos” [25] placed in their uterus. The embryos are in a ‘liminal stage’ while being transferred to their uterus and their liminality [26] continues for two weeks after the ET till a pregnancy test can confirm the outcome of the process. The process of ET thus can be seen as a ‘rite of passage’ [27] for the embryos to ‘become a thing’ or a ‘being’ [28, 29]. ARTs break down the traditional linearity of reproduction into a series of simultaneous and discontinuous steps where each step is an end in itself that contribute to the technology aided pregnancy as a whole [30]. For the next 15 days their corporeal being goes into an ambiguous situation. An ex-surrogate Rama in Delhi while narrating about her experience after the ET mentioned that:

*“…I didn’t feel any difference. I returned home in the same condition in which I came here…”,*

This clearly reflects that the period which follows the ET, the surrogates are not pregnant but are neither ‘themselves’. Rama further goes on to saying that:

*“…but I have to take care since if I do not eat well and rest well, the conception will not take place…” which reflects her sense of awareness and responsibility towards an expected entity.*

Therefore, it can be said that after ET, the surrogates are aware that ‘a foreign thing’ has been placed in their body, which has to be nurtured to life/existence. Their body is seen as a site of hope - a passage for fulfillment of the intended parents’ dreams and that of themselves. Their liminal embodiment is realised by the restrictions, hopes and prayers that are entrusted on their body and the attention, special arrangements as well as facilities that it seeks. However, in case of a missed conception, the narrative around surrogacy does not capture the fact that the surrogate has lost out on anything of their ‘own’ in the process. Instead, only the intended parents are sympathised with by the agents and the medical staff. While talking about the loss that these failed conceptions cause for the actors involved, a clinical staff in Delhi Sonam said that: *“… We feel bad for the intended parents. The surrogate doesn’t lose anything. She loses her chance [to earn] though…”*. The task of the surrogate as per the clinical discourse begins at gestating a conception and being unable to do so is seen as a mere loss of an opportunity. Since a missed pregnancy does not result in the end or absence of any pre-existing ‘material’ entity but a liminal entity, the prevalent norms of the surrogacy industry does not expect the surrogate to grieve. Materiality as Hall [31] suggests is produced via ‘certain forms of looking and seeing’ and the absence of any material base that might have gone missing makes surrogates’ loss unnoticeable and makes it difficult for them to justify their grief. But attachment to this ‘liminal entity’ or ‘thing’ that they were eagerly waiting for ‘to come into being’ inside their womb or embody and gestate causes them immense grief. “Thing” as Tim Ingold has pointed out “is a going on, or better, a place where several going ons become entwined… the thing has character not of an externally bounded entity, set over and against the world, but of a knot whose constituent threads, far from being contained within it, trail beyond, only to become caught with other threads in other knots.” [32]. The surrogate’s loss resulting out of the loss of this “thing” is neither external nor corporeal. They embody this loss by embracing the liminality, a decision that they made while deciding to take up the risk of becoming a surrogate. Most surrogates cry at the news. Their crying is taken by the agents and the medical staff as their naivety, their inability to understand and grapple with the technological sophistications. As a surrogate home staff Pragya describes: *“… they cry… they are uneducated and they do not understand much*…” Hence we would like to point out that the grief of the surrogates is ‘disenfranchised’ [33] since it is neither recognised nor socially validated.

### Comprehending the loss

The surrogates think of the procedure of technological intervention and conceptions as something beyond their traditional understanding of pregnancy. Surrogate pregnancies are mostly referred as “kids born through machines” or in their own words - “*machine se bacha hota hai*”. What remains clear for them is the fact that pregnancies are non-sexual and occurs with the aid of “machines” and “medicines”. There is a certain vagueness or ambiguity in the mind of the first-time surrogates about the technological procedures involved in ET. As per the surrogates’ understanding, the embryo transferred into their uterus is merely a ‘thing’ [*jinish/cheez]*, which is also referred to by some as an “egg”. While describing the process of embryo transfer a surrogate in Delhi named Ranju explained that: *“…At the time of transfer, the egg is taken from the party’s body and transferred to mine….”* Another surrogate named Nandini in Kolkata said that:

*“…I was told that the thing with which you conceive a child…. that stuff will be taken from someone else’s body that cannot have a child and will be given to your body. You will have to then just carry the child for 9 months, give birth to it and hand it over to them. This is a noble deed [punyer kaaj]*…”

They understand the technological intervention and the embryo in terms of their “local moral worlds” [34] based on what has been verbally explained to them by agents or fellow surrogates.^7^ Their understanding modifies as they start experiencing the procedure of surrogacy themselves. Yet the technological undertakings upon their bodies remain outside their purview of knowledge.

Unlike in the U.S.A. or Israel, there is no bonding or inter-personal relationship between the intended parents and the surrogates in India [3, 35]. Instead their relationship in most instances is rather distant.^8^ Hence, the surrogates do not have the opportunity to make sense of their pregnancies based on any exchange of hopes, desires or dreams with the intended parents. As a result, the intended parents and the surrogates fail to connect to each other’s ongoing anxieties and emotions. Nonetheless, the surrogates feel responsible to provide them with a child as they have been conditioned to such thinking by the agents and the clinical staff during their recruitment. A surrogate in Kolkata named Myna who was undergoing hormonal stimulations for an embryo transfer expressed her sympathetic feelings towards the intended parents: *“… Poor fellows! They came once… I pray to god so that am able to provide them with their child…”.* Most of the interviewed surrogates found their own ways of making sense of their roles and construct meanings of their actions accordingly. It was observed that both pregnant as well as intending surrogates consider a surrogate child or foetus as “not theirs” and hence belonging to that of the intended parents. The ex-surrogates on the other hand mostly admit to remembering and praying for “that child” that they have relinquished, reflecting their culturally shaped kinship understandings^9^. Their losses similarly get constructed based on their own cultural understandings and positioning within the surrogacy discourse. A very common statement which came from several surrogates who have undergone a failed conception after an ET was that “…*Mera bacha ruka nahi*” [“I was unable to conceive a child”]. Referring to the embryo after conception as a ‘*bacha*’ or ‘child’ reflects the cultural understanding of ‘life at conception’,^10^ also implying that what has been lost preconception is ‘not’ a child. But then again some surrogates who experienced failed conceptions at the ET stage at times tried consoling themselves by saying: *“…it must be her eggs that were bad…”. * Being unable to make a clear sense of what caused a failed conception, they further entrust their grief upon their physical pain. Some surrogates shared that they felt even more disheartened after an unsuccessful pregnancy conception due to the physical pain that they underwent during injection of the painful progesterone-based vaginal shots. They narrated of having experienced “pain” or “*dard/kosto*”^11^ as a result of their loss. By using the word *dard/kosto* in vernacular for pain, the surrogates address the physical pain that they undergo in preparation of their bodies for the ET; as well as for the emotional distress that they experience due to their failure. Not having received any support from the providers of surrogacy to articulate and cope with the loss that they embody and its resulting grief, the surrogates are pushed towards silence [36]. Those staying in surrogate homes and the others during random meetings with other fellow surrogates, often discuss and share their ‘failure’ to conceive. They do this also with their husbands. But they fail to receive any clear answer(s) to their ambiguities regarding what has been lost.

When reflecting on the discipline of disability studies, Judith Butler mentioned that the idea of embodiment almost cannot be conceived without understanding the underlying norms that define them. Hence it is crucial to question and redefine those very norms which determine the subjective experiences of a body since norms validate ideas around a ‘desirable’ body or embodied experience [37, 38]. The established norms even within the ART discourse consider that the task of gestation of the surrogate begin only at conception of a surrogate pregnancy and fail to recognise the ‘liminal embodiment’ of the surrogates. Their liminally embodied subject position along with its affects, attachments and hopes become a ‘non-normative embodiment’ which is not intelligible to the popular understanding [36]. What that needs to be understood is the idea that surrogates become attached to the ‘thing’ that they embody after an ET, due to the hope it offers to their lives. Such hopes for a better future along with the awareness regarding the presence of a foreign entity within ones uterus, re-inscribes their embodiment. During a regular non-assisted pregnancy, a woman becomes aware of her ‘pregnant status’ or her embodiment of another ‘thing’ or entity only at her conception. Unlike regular pregnancy, a surrogate pregnancy is preceded by a period of ‘making’ or transition after an embryo transfer. During this period, the surrogate undergoes the experience of embodying an entity without having any parallels available for making sense of its ‘relation’ since the entity in question or the embryo in itself, is also in its transition. Her attachment to this phase is not necessarily a maternal-foetal attachment but is rather a ‘liminal attachment’ defined by hopes, imaginations and materiality which when absent creates a sense of loss and evokes grief.

The surrogates cannot openly acknowledge nor publicly mourn this loss, as doing so might be taken as their emotional attachment to an expected entity and would dismiss all their future opportunities of being a surrogate. An intended father who had changed his surrogate for a second cycle of embryo transfer^12^ when asked his reason for doing the same mentioned that:

*“...I did not find that lady [previous surrogate] right. Her words and demeanor was not very convincing after the unsuccessful conception. After trying out so many ways [for pregnancy], we do not desire any problem in future…”*

﻿This reflects how slightest of doubt about the nature or intent of the surrogate can push the intended parents to change their surrogate despite the fact that doing so might bring them a long waiting period until they find a suitable surrogate once again. The surrogates thus have to abide by the unsaid norms of the industry. They at best can articulate their failures about a ‘task’ in hand and look forward towards another opportunity to conceive a successful surrogate pregnancy.

### Yet another loss

Most of these intended parents, opt for surrogacy as their last resort towards having a genetically-related child. This decision is only taken after undergoing unsuccessful fertility treatment for several years and when all other means of ARTs are exhausted. Therefore, the missed attempts of conception during surrogacy come as another setback and add to their sense of loss. An intended father named Kumar in Delhi, while talking about his decision to opt for surrogacy, mentioned that:

*“… Everyone keeps asking her [his wife] the reason why we are not having a child. It affects her. With every failed cycle she feels even more miserable. It affects me too [starts crying] …my work gets affected. We have everything but not the main thing [child]…”*

Therefore, it can be said that this loss due to a missed surrogacy conception is non-finite [39] in its nature since its adds on to their ongoing struggle of dealing with childlessness. However, their experience at failed surrogate conception differs from all other previous reproductive failures mainly due to the disembodied nature of the loss for them [36]. Unlike the surrogates who feel very confident about their reproductive capacities, the intended parents feel extremely vulnerable and anxious during the whole procedure for not having any control over their attempted conception. They feel *“there is hardly anything within their hands”* and leave everything in *“the hands of the doctor and the agent”.* Some of the intended parents experience a chronic sense of sorrow and depression due to infertility and an event of a failed surrogate conception further add to their grief. In addition, a series of reproductive disruptions often create an adverse effect on the interpersonal relationship of the intended parents. An intended mother named Jyoti in Delhi who was shattered and depressed after three failed IVF cycles, including the resulting distance from her husband said that:﻿

*“…He has not been speaking to me for almost three weeks since our last IVF attempt. I don’t know what is going on in his head. He doesn’t share…”*

Therefore, the loss and frustration resulting out of a missed conception further shatters the intended parents. Moreover, the involvement of the extended family which is typical of the Indian scenario makes things tough for the couple. An intended father in his mid-forties named Arvind shared that:

*“… Childlessness has been causing a lot of discord and altercation between us. But then I can understand since I stay away for my work and she has nothing to herself. Two of her sister-in-laws live in the same house and each of them have a child. …. Every time I face a failure with assisted reproductive technologies; I gain my strength back thinking of my wife…”*

The intended parents try to be supportive towards each other to the extent possible. However, we noticed successive failed attempts whether during own IVFs or surrogate conceptions also opens up a series of mutual blame game between the couples, leading to marital discords. Literature on (in)fertility talks about the typical grief responses, loss of self-esteem and depression which persons undergoing involuntary childlessness might encounter (refer to [40–42]). Often, the intended fathers start blaming their wives for their egg quality and lifestyle, adding yet another unpleasant dimension to their pre-existing loss and grief. Jyoti's husband Manish duirng their consultation complained that:

*“… I go for jogging and to the gym every single day. I try to wake her up everyday but she refuses to come along. How will the quality of her eggs improve like this? Her IQ is so poor. The IQ of her entire family is nil…For the next attempt, I have asked for donor eggs…”*

This also sheds light on the patriarchal forces that are at work within the Indian society which re-surfaces during reproductive decision-making.^13^ Reproductive losses resulting out of failed ET of the surrogate, is only a single instance that triggers its surfacing.

### No space to grieve

We noticed that even though such a loss of the intended parents is recognised by the medical discourse, their grieving is not given much space within the clinical discussions. Since such events are considered commonplace due to lack of absolute reliability of ARTs, the couples are instead encouraged to keep their optimistic spirits high and stay positive for their next try. During one such counselling sessions after a failed conception, a fertility specialist, Dr. Singh told the intended parents:

*“…Don’t feel disheartened. Couples keep trying for several years. You still have two more cycles left with this surrogate. The surrogate (body) is good. It may click this time…”*

Several hours of (informal) counselling, optimistic reassurances, persuasion for patience and stories of success do not allow the intended parents’ grief to be realised. Some take time off from the rigorous and demanding process of IVF and get back after a year or two. But most continue with the process immediately after a month or at least within three months to avoid going through the hassle of finding another surrogate since their contracts allow them an option of attempting three IVF cycles with the same surrogate within a period of one year. This keeps the intended parents tied up with the rigorous process of organising and re-planning without having reflected upon if at all they are ready to take another chance. Also, some intended parents at times take such missed attempts or failures as inevitable part of their surrogacy journey. Thus, despite experiencing a deep sense of loss and failure, they subscribe to the larger surrogacy narrative of just accepting them and moving on. Further, fertility ‘treatment’ is a private decision, and most, if not all, couples seldom share their involvement in the same with their friends and relatives [14]. This is done mainly to avoid the stigma attached with infertility in general, and surrogacy in particular.

With the clinics not ready to accommodate their grieving and for most, if not all, the extended family and friends being kept out of the purview of discussion, the intended parents neither have a physical space to grieve, nor do they have the luxury to space out their attempts. The latter is mainly due to two reasons, first, the intended parents’ urgent desire and continuous social pressure to keep trying for a child; and second, their surrogacy contracts being time-bound. Although, some intended parents confide in close friends or parents, we found that most do not have the luxury to find solace somewhere else. As a result, the intended parents have to deal with their loss(es) all by themselves without being sure about its exact nature and extent. Also, there is a certain degree of ambiguity in their minds regarding what their loss actually is. “When a loss combines with ambiguity, there is no closure and the rupture continues until a perceptual shift restores relations, meanings, and hope” ([43]﻿﻿﻿﻿ p. ﻿108). The intended parents thus try to cope with their disenfranchised grief [33] and wait for a closure by looking forward to the successful conception and birth of a surrogate child in their next attempt.

### Ethical considerations: Can there be a better way for dealing with these losses?

At the end of an interview in Kolkata, an intended father Arvind, who has been trying surrogacy (with long breaks inbetween) since past seven years remarked:

*“… It was nice talking to you… being able to share these things with you. One cannot talk about these things with anybody. My wife is perpetually depressed. I cannot pour myself out in front of her. I am feeling much lighter now after talking to you…”*

As we have mentioned, missed pregnancy conceptions or failed conceptions are common during surrogacy in India; and most intended parents, especially the intended fathers, despite being aggrieved do not have a space to share or grieve their losses resulting out of such missed attempts of conception. These experiences made us realise that the preconception losses that the surrogates and the intended parents undergo during the course of surrogacy need to be re-considered within an ethical and practical framework. The empirical data has indicated impressively that the current situation has more ethical and affective dimension, than just the overall ethical-legal question pertaining to whether surrogacy should be allowed or not. In countries where commercial surrogacy is practiced (with or without legal regulations), like that in India, Mexico, USA, Russia, Ukraine, Georgia, it is necessary to create better spaces for the sharing of grief and pain. An arrangement of commercial surrogacy revolves around the ongoing experiences of mutuality and dependency between the actors themselves; including the market and the technology that shape their expectations. Despite these entanglements of roles and experiences within the surrogacy arrangement, the inter-personal relationships between the intended parents and surrogates, and that between the surrogates and doctors are very distant - with almost no space for sharing of feelings, emotions, expectations or anxieties with each other. Our study revealed that although local intended parents are more in touch with their surrogates as compared to their transnational counterparts, not all of them choose to stay in regular contact with the surrogates. While overseas couples and especially persons of foreign origins seldom get to meet, connect or interact with their surrogates due to linguistic, legal, spatio-temporal, racial and class barriers,^14^ the locals and NRIs who might not be facing some of the above barriers are usually advised by the clinics and agencies to maintain a minimum interpersonal contact with their surrogates. Such advices are offered in the pretext of concealing their participation in surrogacy and dismissing any chances of their surrogates developing any out of term emotional and financial expectation from them. Since most intended parents of Indian origin wish to conceal their participation in surrogacy, the agencies and clinics present this prerequisite of maintaining distance from their surrogate to the intended parents, as the first step towards achieving such confidentiality. However, as noticed, over the years such advices towards safeguarding confidentiality as imparted by the clinics have rather become a strategy to exert their own importance as mediators and less of a demand from the intended parents themselves. While we found that some intended parents choose to keep direct contact with the surrogates during the nine months of pregnancy, their conversations if any, are very functional and often mediated by agents or clinical staff during the preconception stage, until the ET. We found that till the day of the ET, most intended parents meet the surrogate only once and their meetings last for only few minutes with agents, clinical staff and others leaving them with little or no scope for personal sharing and/or bonding.

Ell y Teman [35] while studying surrogacy in Israel has emphasised on the importance of interpersonal relationships between the actors for an arrangement to succeed. In addition, we would like to follow Carmel Shalev’s suggestion where she proposes to understand surrogacy as an arrangement involving a particular moral responsibility on the part of all the actors concerned [44]. This is because unlike other types of commercial contracts such as buying a car, surrogacy involves the act of bringing a new life into existence and it requires its actors to be responsible for the wellbeing of this new expected entity, as well as that of each other. This normative scenario stands in contrast to the current situation, where the whole procedure is loaded with anxiety, uncertainty and spontaneous decision-makings, undermining often the explicit addressal of moral expectations. Instead, surrogates and intended parents suffer from bouts of blaming, shaming, sense of inadequacy and moral resignation.

The lack of any direct interpersonal relationship between the actors keeps them from sharing each other’s concerns and in shouldering their moral responsibility. When we mean ‘moral responsibility’, we reject any reductionist view that responsibility is only a moral practice of blaming and guilt ascription, as sometimes conceptualised in medical sociology (e.g. [45, 46]). Instead, moral responsibility is understood as a basic form to understand and describe folk moral language (see for theoretical details: [47]). It is construed as meta-ethical construction of morality based on three major underlying assumptions: First, of a specific relationship between a moral subject and a moral object (e.g. mother and child, intended parents and surrogate, doctor and patient). Second, a time frame that is forward or backward directed and is based on the roles, functions or causal conditions. And third, a concrete normative assumption how the moral orientation as well as the act of the subject toward the object ascribed is justified. If this justification is built on virtue ethics, we say that X feels responsible because she cares for Y. If this justification is built on a hierarchal or legal function, we say that X is responsible for Y because he is in charge of the decision. If this justification is built on universal rights, we say X is responsible for not harming Y because he respects the universal right to life and bodily integrity. In the legal and professional sphere, the backwards type of responsibility prevails (e.g. X is responsible for killing Y and therefore is guilty). Instead, in the social and political sphere the forward type of moral responsibility is important to ensure moral motivation and to build moral trust in social relationships (see [48]) (e.g. X is responsible to protect the child against any harm and to give him the best education).

The latter form of forward-directed moral responsibility, as we would like to argue, is helpful to address the actor’s concrete relationship in the context of commercial surrogacy. In order for that to happen, the actors during commercial surrogacy need to understand that they owe each other an inter-personal relationship, which is more open, frank and sincere. Some feminist ethics approaches have shown that close-knit relational interaction between actors is a normative background assumption. It is based on the insight that humans are not isolated but always in a relationship [49]. Such a relational assumption has implications for a moral analysis of existing situations, as well as for the proposed solution based on this research [50]. But it is also important to reflect upon the two dimensions of this complex setting; one, involving the relationship between the professionals and non-professionals, i.e. the relationship of the fertility doctors with the surrogates and intended parents; and the other, involving the relationship between the two lay actors viz. the surrogates and the intended parents, to be able to understand the varied experiences and expectations of each of the actors from the other. The nature and scope of these two sets of relationships needs to be understood differently.

We suggest to refer to a normative approach of *an ethics of care* [51] for the justification of moral responsibility between the intended parents and the surrogate. By this we hope to contribute towards a better understanding of the ongoing encounters between both parties. Care involves attending to their feelings, needs, desires and thoughts of those cared for, and honing the skills towards understanding a situation from the other person’s point of view [51, 52]. In addition, it has the potential to address the processual anxieties, failures and experiences of uncertainties of both the surrogates as well as the intended parents and provide them with space to grieve, share and attain closures. Studies on surrogacy in the U.S. indicate that since the surrogates share a personal relationship with the intended parents, they are able to articulate their losses as a failure to give a baby to the intended parents [3]. Lack of any such relationship between the surrogates and intended parents in India keeps them from constructing such meaning of their losses. Despite large socio-economic differences between the surrogates in the US and in India, allowing space for close inter-personal relationships should be considered as an alternative to the present problem of surrogates suffering alone in silence, at least from a professional medical ethical point of view and needs to be adapted to the Indian context. But how can such a relationship be fostered or encouraged? Held has noted that, “the social relations in whom the persons are enmeshed constitute their personhood” ([51] p. 101). Not only do these social relations provide the actors with the space to constitute themselves as individuals but also with the impartial rules for treating each other with equal concern and respect [51]. During the course of commercial surrogacy, the framework of an ‘ethics of care’ helps us to understand the intensity of differential labour performed by the different actors, the role of affect in its shaping and the consequent experiences of loss at its disruption. This clearly shows that “much that has moral value in both personal and political life is “beyond justice” ([51] p. 102). According to Held, justice or deontological considerations of right and duties are not wrong or irrelevant, but do not determine any moral relationship.

So, the surrogates can best be cared for by the intended mother or both the intended parents since it is for them that they are going to gestate a child. Studies on surrogacy from around the world [1, 35, 53] have indicated that close contact between the surrogates and the intended mothers enable them to help each other out with the experience of pregnancy and bonding with the foetus. A major barrier between most intended parents of Indian origin and the surrogates that prevent them from bonding is the felt need amongst most intended parents’ to maintain secrecy around their surrogacy arrangement. Such a need in the Indian context stems from intimately linking “the cultural “imaginings” of visible social triad of mother/father/child with an invisible biological triangle of womb, semen and foetus” [54]. Therefore, couples opting for surrogacy aim to re-create a culturally expected “visuality of fertility” [54] by invisibilising their infertility and hence recreating the visible social triad in their lives. To avoid future disclosure of their surrogacy arrangement to the child or even to the extended family, intended parents often keep a check on the level of intimacy with the surrogate and plan to severe off all ties post-delivery. Another reason that keeps them from bonding is their class bias. The very strong cultural heritage of caste/class hierarchy that perpetuates within the Indian and especially the Hindu society (refer to [55])^15^ prevents emergence of any kind of intimacy and confines their relationship to a distant and utilitarian one. Their class prejudices are further perpetuated by the involvement of the mediators or the surrogacy agents who widen these distances by warning the intended parents about future implications of any intimacies with their surrogates- in the form of disclosure leading to destabilising family relations or facing blackmailing from surrogates and her family for financial gains. By doing so, the agents and agencies justify their own mediating positions. However, during this study, we found that on occasions where the intended parents decide to closely engage with their surrogates [mainly when a surrogate agency and/or home is not involved], the resulting relationships as reported mainly by the surrogates have been extremely intimate and supportive, suggesting how fruitful these close relationships can be. The intended parents therefore should set aside their class-prejudices to be able to develop some empathy for the surrogates and take up their responsibility to care. By doing so, they themselves might feel much more connected to the process.

But how do we describe and assess the relationship between doctors on one side and intended parents and surrogates on the other? Here, an ethics of care position is too weak because doctors have always professional duty to care for their patients, which includes also the surrogates given the embodied risk they take. Since intended parents and surrogates invest a lot in terms of their emotions, by taking risks and stretching their socio-economic boundaries, it then becomes the professional duty of the fertility doctors to protect both sets of clients equally without providing preferential treatment to either. The whole procedure becomes very existential and demanding for both the surrogates and the intended parents, which in turn makes them vulnerable in their own ways. Vulnerability here refers to a context-related risk for a person due to particular power relations, to be harmed (physically or psychologically) or disrespected with regard to their self-determination [56, 57].

Although being relatively well-informed and in a better position to negotiate, the intended parents still feel considerably vulnerable because of the uncertainty involved in the procedure. Further, the decision to make use of ART implies that the intended parents need to place their sexual and reproductive lives up for scrutiny. This, however, is not a conventional practice in India since such details are mostly considered as private and often sharing of the same is even tabooed for Indian couples. Entrusting the clinics and the agents with those personal details which the couple might have not shared with any other close confidant; the couples feel very dependent on them, especially on the fertility doctors and start having expectations for emotional support from them. Again, for some intended parents surrogacy also becomes an attempt to save their marriage since procreation is not just an innate desire but also a social duty of these Indian married couples and a key expectation of each other from their marriage. Such couples feel very vulnerable and insecure during the whole procedure for not being in charge of the entire process. Such vulnerabilities of the actors can be countered by the actors themselves (intended parents, the surrogates and the doctors) by improving their inter-personal relationships, since ethics of care requires forging and nurturing of such relationships. The logic underlying this ethics of care needs to be seen as a psychological logic of relationships [58]. As such, an ethics of care is very appropriate to cover such a context, while an ethics of rights and duties often oversees the psychological, emotional and social constraints of such relationships.

The problem of professional medical ethics during commercial surrogacy often arises not since information is not provided to the surrogates, but rather due to the way in which information is communicated. Explaining the medical and legal procedure to the surrogates in a localised term along with selective communication of risks and their rights often falls short of protecting the surrogate’s rights and interests. Although as per our experience, most fertility specialists provide personal time and counselling to the intended parents, their attempt remains to respect the moral views of the intended parents. Further beyond a particular point, the doctors step aside from matters of interpersonal engagements and mundane coordination between the intended parents and the surrogates and let the agents or agencies take charge. In the Indian context, like in most western contexts, since the doctors are offered a superior position by patients, doctors can make use of the hierarchy in a positive way and encourage increasing interactions and communication between surrogates and the intended parents. Despite a cultural need to maintain secrecy due to the stigma around infertility and adoption, the medical professionals are the ones who are in the position to become the torch bearers to de-stigmatise surrogacy and enable openness around the process. Therefore, it is the doctors who need to take up the professional moral responsibility to enhance intimacy, care and understanding between the surrogates and intended parents as well as provide best medical and psychological care for both parties. While the surrogates and the intended parents feel convinced and justify the need for their rightful participation in the practice of gestational surrogacy, their sense of commitment towards the practice can be realised by nurturing an ethics of care. Not mentioning the moral dimension by neglecting it or seeing it as per economical contract, can lead to rather implicit unclear moral dilemmas and even moral distress. Therefore, to nurture caring relationships, we agree with Beier [59] that surrogacy needs to be understood as a moral commitment by its key actors. It cannot be a one-sided commitment, but has to have elements of mutuality. Commercial surrogacy needs to be undertaken as a relational process, which can only be sustained by care and sharing of interpersonal concerns. To complete this thread of mutuality and care, we think that the role of the surrogate agencies that function as the brokers between the doctors, intended parents and surrogates also needs to be legally regulated and rather limited. How their roles can be granted recognition deserves a different discussion and does not fall within the scope of this paper. But legally specifying their roles and granting the doctors and their clinics an upper hand over the agencies can work to nullify their attempts towards distancing the surrogates and the intended parents.

- Failed attempts to surrogate pregnancies or missed conceptions are not considered as events of any significance within the surrogacy discourse.
- The process of embryo transfers is a rite of passage for the embryos to become a ‘being’ from a ‘thing’.
- During the waiting period between the embryo transfers until the pregnancy confirmatory test, the surrogates have a liminal embodiment.
- Absence of any pre-existing material entity fails to capture the loss of the surrogates resulting out of a missed conception.
- As a result, the surrogates experience an ambiguous or uncertain loss and a disenfranchised grief.
- The intended parents experience the failed surrogate conceptions as a disembodied loss which adds on to their previously experienced losses and takes the form of a chronic sorrow.
- The loss of the intended parents is recognised but their grief not given space.
- Commercial surrogacy is a relational process, but lack of interpersonal relationship keeps actors from sharing each other’s concerns and constructing the meaning of each other’s losses.
- Improved communication and better support system might help to prepare actors in dealing with risks and losses.

## Conclusions

To conclude, we would like to state that the surrogates and the intended parents experience different variants and degrees of losses when faced with events of missed or failed conceptions. Since both the surrogates and the intended parents put forth considerable ‘labour’ at the preconception stage, a failure to conceive leads to an experience of loss for them. Therefore, to understand preconception losses, it becomes important to take into account the physical, emotional and psycho-social needs of the actors participating in these surrogate pregnancies along with their vulnerability. In this paper, we have discussed the struggle of the surrogates in comprehending their losses resulting out of their missed conceptions as well as the impact of the same upon the intended parents. By analysing the causality of such losses, its bearing and impact upon the actors, we presented the need for giving due attention and space to these preconception losses which are often left unattended. A new surrogacy bill^16^ which is being drafted is likely to ensure ways of informing the surrogates on the complete medical procedure involved rather than the present tokenistic practice of signing a contract. The rights of both the surrogates and the intended parents to counselling and support needs to find its place within this new bill since it plays a crucial role in shaping the entire surrogacy discourse. It is alarming that these preconception failures and disruptions are rather neglected in the ethical, medical and public discourse. Although these experience of preconception losses are not same as that of a post-conception pregnancy loss, we would like to argue that these ‘reproductive disruptions’ that occur due to the failure of assisted reproductive technology, needs to be seen as reproductive losses because of the immediate impact it creates in the lives of the actors involved. By analysing the experiences, expectations and the nature of relationships between the actors during commercial (gestational) surrogacy, we reconstructed different stages of their continuous process of loss and their inability to grieve. Thus clearly the emotional experiences of surrogates and intended parents contravene the professional/public conceptualisation of failed attempts to conceive a pregnancy as ‘non-events’. Hence we argue that these failed or missed attempts to pregnancy needs to be offered its due place and recognition within the discourse of commercial surrogacy which can become possible only when the preconception stages like the rest of the procedure are legally regulated. Regulatory guidelines can bestow the doctors with professional obligations to step in and offer thorough counselling and support to the intended parents on events of such losses. On the other hand, if the intended parents and their surrogates are ensured direct contact at the time of recruitment and preconception preparations, as their rights rather than privilege, it can potentially provide them the opportunity to develop a moral commitment towards each other and mutually construct meanings of their fears. Doing so might help prepare both the actors and especially the surrogates, in dealing with risks and losses. Having said this, we would like to point out that although such rights to engage with the intended parents might empower the surrogates, the structural inequalities that are a part of the Indian society might continue to keep the intended parents distant from their surrogates by reducing their rights to rather formal obligations. However, our suggestions can only be the first step towards improving upon the scope for any preconceptions engagements and arrangements in place. This prompts us to rather stress that there is an increasing need to study the preconception preparatory stage of a surrogacy arrangement until the embryo transfers in more detail and analyse the long-term impacts of these preconception disruptions on the actors involved.

## Abbreviations

ET: Embryo transfer
NCR: National Capital Region

## Footnotes

1 Mitra S, Unpublished Manuscript. Postconception disruptions during surrogacy: end of a beginning?

2 In India as per the ICMR Guidelines, a surrogate needs to be a married heterosexual woman with previous experience of childbirth of her own. Her husband becomes her legal guardian who is required to be a party to her surrogacy contract and relinquish all claims of paternity.

3 Some surrogates are not informed about their pregnancies without this confirmatory scan to avoid later setbacks.

4 The National Capital Region (NCR) in India is the designation for the conurbation or metropolitan area which encompasses the entire National Capital Territory of Delhi, which includes New Delhi, as well as urban areas surrounding it in neighboring states of Haryana, Uttar Pradesh and Rajasthan (Wikipedia).

5 Since 2013 as per a directive issued by the Ministry of Home Affirs, India closed down its market of commercial surrogacy for homosexual couples or single parents. Refer to http://www.queerty.com/indian-government-bans-foreign-gay-couples-from-using-surrogates-20130118, accessed on 2nd Oct. 2015.

6 All information sheets and consent forms were translated from English to Hindi and Bangla. Where participants were unable to read, the information sheet and consent form was read out to her/him in her/his preferred language and consent was obtained.

7 They do not receive any documents or manual about surrogacy. Everything is explained verbally. The surrogacy contract which they sign on the day of the embryo transfer is in English and employs technical legal language which is not explained to the surrogate.

8 The relationships between the surrogates and their intended parents as noticed during this study, is mostly mediated by local agents and agencies till the embryo transfer. We found that it is only during the pregnancy phase or post-conception phase that some intended parents keep direct contact with their surrogates. Again on certain rare occasion, surrogates might be completely clueless regarding who their intended parents are on grounds of ensuring secrecy and granting the intended parents anonymity.

9 Pande [4] has talked about how surrogates give importance to their own body fluids like blood and sweat over genes to establish their claim over their surrogate child

10 According to the *Caraka Samhita*, a Hindu medical text, the soul is already joined with matter in the act of conception. The soul is described as descending “…into the union of semen and (menstrual) blood in the womb in keeping with the (karmically produced) psychic disposition (of the embryonic matter)” [60].

11 Dard is Hindi and kosto in Bengali means pain

12 Surrogates in India are usually bound by contract to undergo a minimum of three cycles of embryo transfer for the same set of intended parents, be their previous cycles result in failed conceptions. They are paid separately for each attempted transfer.

13 Similar instances of patriarchy can be noticed during the relative willingness of the couples to go with donor oocytes whereas instant opposition towards accepting donor sperm, even if they later settle for the same.

14 Intended parents of foreign origins would usually visit India twice- one during commissioning the surrogacy and once nearer to or after the date of the delivery. The locally based intended parents as noticed during this study on the other hand frequently get to cross paths with their surrogates during their regular checkups especially until the embryo transfer and pregnancy confirmation.

15 K.L. Sharma said that caste has been inhered in class and class inhered in caste in the Indian society and continues to have their inseparable mix even till this day.

16 The Draft Assisted Reproductive Technology Bill 2014 is out and in under review of the government and policy circles for further changes. A new version of this draft bill is on its way.
